# Immune Checkpoint Inhibitors and Chemoradiation for Limited-Stage Small Cell Lung Cancer

**DOI:** 10.1007/s11864-022-00989-7

**Published:** 2022-06-18

**Authors:** Brian Schlick, Misty Dawn Shields, Julian A. Marin-Acevedo, Ishika Patel, Bruna Pellini

**Affiliations:** 1 Department of Oncologic Sciences, Morsani College of Medicine, University of South Florida, 12902 Magnolia Dr, GME Office, Tampa, FL 33612 USA; 2 Department of Public Health, University of South Florida, 4202 E Fowler Ave, Tampa, FL 33620 USA; 3 Department of Thoracic Oncology, Moffitt Cancer Center and Research Institute, 12902 Magnolia Dr, CSB 6-THOR PROG, Tampa, FL 33612 USA

**Keywords:** Limited-stage small cell lung cancer, LS-SCLC, Immunotherapy, Concurrent immunotherapy chemoradiation, Immune checkpoint inhibitors

## Abstract

Limited-stage small cell lung cancer (LS-SCLC) is a potentially curable disease. However, most patients develop disease relapse shortly after definitive treatment. The landmark trials IMpower133 and CASPIAN demonstrated a survival benefit with the addition of immunotherapy to first-line platinum/etoposide for extensive-stage small cell lung cancer. Therefore, it is critical to determine whether advancements in overall survival with immunotherapy can be translated earlier into the treatment paradigm for LS-SCLC. Decades of robust preclinical research into the synergism of radiation therapy and immunotherapy set the stage for the combination of these treatment modalities. Recently published data suggests tolerability of single agent immunotherapy concurrent with chemoradiation in LS-SCLC, along with promising efficacy. However, combination immunotherapy in the consolidation setting appears too toxic, although this may be reflective of the dosing schedule rather than inherent to any combination immune checkpoint blockade. Here, we review underlying mechanisms of synergy with the combination of radiation and immunotherapy, the safety and efficacy of respective treatment modalities, and the ongoing trials that are exploring novel therapeutic approaches for LS-SCLC. Pivotal trials in LS-SCLC are ongoing and anticipated to aid in understanding efficacy and safety of immunotherapy with concurrent platinum-based chemoradiotherapy.

## Introduction

Small cell lung cancer (SCLC) is an aggressive malignancy with recalcitrant neuroendocrine features and poor five-year overall survival [1]. The current standard of care (SoC) for limited-stage SCLC (LS-SCLC), defined as disease confined to a single radiation field, is concurrent chemoradiation (CCRT) with a platinum analog plus etoposide [2–5]. Patients with interval disease response to CCRT are candidates for prophylactic cranial radiation (PCI) for an added survival benefit of 5.4% at three years [6, 7]. Although LS-SCLC is potentially curable with definitive chemoradiation, outcomes remain poor with a median progression-free-survival (PFS) and median overall survival (OS) of 15 months and 30 months, respectively [8]. Since CCRT became the SoC in the 1990s,[9, 10] minimal changes have been adopted into this therapeutic algorithm.

Similarly, the use of chemotherapy alone was standard for patients with extensive-stage SCLC (ES-SCLC) until the results of two landmark trials, IMpower-133 and CASPIAN, were published in 2018 and 2019 leading to the respective FDA approval of the anti-PD-L1 immune checkpoint inhibitors (ICIs) atezolizumab and durvalumab in combination with platinum/etoposide in the first-line setting [11, 12]. The implementation of immunotherapy in the first-line setting for ES-SCLC has improved median overall survival by 2-3 months [11, 12]. Whether the addition of ICIs to CCRT will result in similar benefits remains to be proven. Here, we review the preclinical evidence for combining radiation and immunotherapy, the safety and efficacy of the combination of ICIs with CCRT, and the ongoing trials exploring novel therapeutic approaches for LS-SCLC.

## Immunotherapy and radiation therapy synergism

The combination of radiation therapy and immunotherapy is a promising approach to the treatment of recalcitrant malignancies such as SCLC. In 1979, Steel [13] described mechanisms whereby combination approaches with drugs and radiation synergize by modulating the tumor microenvironment and improving outcomes [14•]. Modernization of the Steel hypothesis in the era of immunotherapy proposed five exploitable mechanisms for interactions between radiation therapy and immunotherapy, 1) spatial cooperation, 2) cytotoxic enhancement, 3) biological cooperation, 4) temporal modulation, and 5) normal tissue protection [15, 16].

**Fig. 1:**
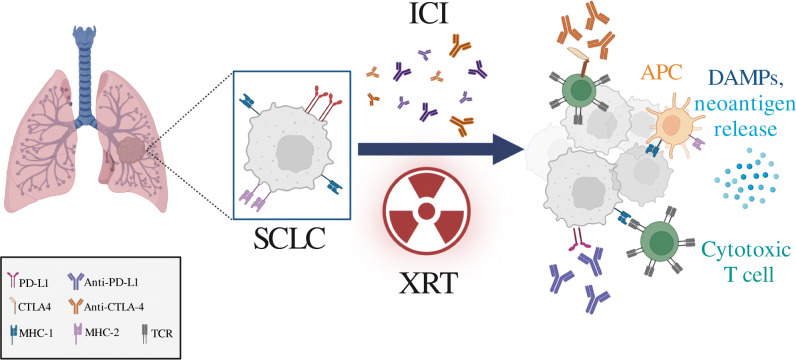
Synergism between immunotherapy and radiation. Radiation causes neoantigen release from cancer cells. Antigen presenting cells prime T cells to these neoantigens, allowing for T cell-mediated cytotoxicity of the cancer cells. Anti-PD-(L)1 and anti-CTLA4 antibodies block these negative regulators on cancer and immune cells. SCLC, small cell lung cancer; XRT, radiation therapy; ICI, immune checkpoint inhibitors; APC, antigen presenting cells; DAMPs, damage-associated molecular patterns.

Radiation therapy may potentiate synchronous immunostimulatory and immunosuppressive effects within a tumor site [14•, 17]. Following radiation therapy, cytosolic DNA generated from radiation-induced double-stranded DNA breaks activate cyclic GMP-AMP synthase (cGAS)-stimulator of interferon genes (STING) pathway [14•, 18]. The STING pathway activates interferon regulatory factor 3 (IRF3), resulting in the production of type I interferons for antigen cross presentation and immune stimulation [14•, 18]. Radiation therapy also enhances protein degradation and intracellular peptide pools that increase T-cell receptor repertoire and diversity [19], increase expression of major histocompatibility complex type I for enhanced antigen presentation [20], and increase FAS (CD95)/FAS ligand (FasL) interactions leading to irradiation-induced cytotoxic T-cell mediated tumor apoptosis [21]. Interestingly, the use of concurrent radiation therapy plus anti-CTLA4 therapies may lead to the upregulation of PD-L1 expression, which is associated with T cell exhaustion and decreased CD8/Treg ratio [19]. These changes favor immunosuppression and tumor resistance to therapy [19]. The use of triple therapy with non-redundant mechanisms (e.g., radiation therapy, anti-CTLA4, and anti-PD-L1) may be necessary to overcome radiation therapy-induced acquired resistance to anti-CTLA4 based therapies (Fig. 1) [19].

In 1953, following focal tumor irradiation, R.H. Mole observed the regression of distant non-irradiated sites within the same organism [22]. As a result, Mole proposed the hypothesis of the “abscopal effect” (from Latin, “ab scopus,” that is, “away from the target”) wherein local radiation therapy may initiate an immune-mediated antitumor response as a crosstalk between local and metastatic sites of disease [22]. For decades, the abscopal effect has remained sporadic, and documented as a poorly understood phenomenon [23, 24]. Demaria et al. [25] demonstrated the abscopal effect is linked to an immune-mediated mechanism. Immunocompetent mice bearing 67NR syngeneic mammary carcinoma on bilateral flanks were treated with ionizing radiation at a single dose (2 or 6 Gy) to only one tumor, with or without concurrent use of Flt3-Ligand (Flt3-L) to enhance dendritic cell activation [25]. Growth delay with radiation therapy occurred as anticipated in the irradiated tumor, however growth delay in the non-irradiated tumor occurred only in the combination of radiation therapy with Flt3-L [25]. Moreover, growth delay was not observed in T-cell deficient *nude* mice after dual treatment with radiation therapy plus Flt3-L, demonstrating T cell recognition is required for abscopal effect [25]. A proof-of-principle phase II study investigated whether the addition of pro-immunogenic granulocyte-macrophage colony-stimulating factor (GM-CSF) through dendritic cell maturation could reproducibly induce immunity-mediated tumor response outside the radiation field [26]. Eligible patients with metastatic breast cancer and other solid tumors who had stable or progressive disease after systemic therapy, with at least three measurable sites of metastatic disease, were enrolled. Radiation was given during systemic therapy to one of the lesions, delivered as 35 Gy in 10 fractions, with GM-CSF. At day 22, radiation was resumed, and the same radiation dose was delivered to a second metastatic site, again with GM-CSF. Abscopal responses in the non-irradiated measurable metastatic sites by positron emission tomography (PET) imaging occurred in 11 of 41 accrued patients (26.8%, 95% CI 14.2–42.9), including patients with breast cancer (n=5), non-small cell lung cancer (n=4), and thymic cancer (n=2) [26]. These combined findings suggest the synergism of radiation therapy and immunotherapy, setting the stage for novel combinations utilizing both treatment modalities.

## Immunotherapy and radiation therapy combination safety

CCRT with cisplatin and etoposide remains SoC for LS-SCLC [2, 8]. This regimen is often associated with side effects such as fatigue, cytopenias, esophagitis, and gastrointestinal toxicities [8]. Serious side effects such as neutropenic fever (18%), severe esophagitis (19%), and severe pneumonitis (2%) may also occur [8]. Although ICIs are not approved for LS-SCLC, triplet therapy with anti-PD-L1 therapies (e.g., atezolizumab or durvalumab) combined with platinum/etoposide are the SoC therapy for ES-SCLC [11, 12]. The combination of platinum/etoposide with ICI is associated with immune-mediated adverse events such as rash (19%), hypothyroidism (9–13%), hepatitis (3–7%), and infusion reactions (6%) [11, 12]. Serious adverse events (grade 3 or higher) including pneumonitis (2–3%) and diarrhea/colitis (1.5–2%) can occur [11, 12]. However, in the therapeutic context of thoracic radiation implemented in strategies for LS-SCLC, and the inherent pulmonary toxicities (e.g., pneumonitis) associated with ICI, the theoretical risk of pneumonitis and pulmonary adverse events may occur at a higher frequency, but remains largely unknown [27]. Therefore, safety assessments within trials with a focus on pulmonary adverse events should be executed, while simultaneously considering the approach and cadence of multimodal concurrent and consolidative strategies for LS-SCLC.

Dual ICIs combination with the anti-PD1, durvalumab, and the anti-CTLA-4, tremelimumab, were investigated in combination with or without induction stereotactic body radiation therapy (SBRT) to one selected tumor site in 18 patients with relapsed or metastatic SCLC in a phase II study (NCT02701400) [28]. Interestingly, the incidence of cough and dyspnea was lower in patients who received SBRT followed by dual ICIs, versus patients who received dual ICIs alone (22% and 11% vs. 33% and 55%, for cough and dyspnea, respectively) [28]. While encouraging, these results are limited by the modest sample size. In contrast to this data, two studies conducted in patients with metastatic NSCLC demonstrated higher rates of pulmonary toxicity with the administration of radiation before ICIs [29, 30]. Shaverdian et al. [29] performed a single institution secondary analysis of 98 patients enrolled into KEYNOTE-001 (NCT01295827), who received radiation therapy prior to trial enrollment. A higher incidence of pulmonary toxicities among patients who received radiation before pembrolizumab was reported, compared to those who received pembrolizumab alone (63% vs. 40%, respectively; *p*=.052). PEMBRO-RT (NCT02492568), a randomized Phase II study investigating the role of pembrolizumab after high dose radiation with SBRT to a single target lesion in advanced NSCLC, reported the incidence of pulmonary toxicities (e.g., pneumonia) was higher in patients who received SBRT prior to pembrolizumab compared to those who received pembrolizumab alone (9 of 35 [26%] vs. 3 of 37 [8%], respectively; *p*= .06) [30]. Conflicting results among these studies may be secondary to underlying heterogeneity and biological differences among histological subtypes of lung cancer (e.g., SCLC vs. NSCLC). Alternatively, these may be explained by variations in sample size, dose schedules, or regimens utilized.

Dual ICI consolidation after CCRT was investigated in STIMULI, a randomized phase II clinical trial for LS-SCLC (NCT02046733) [31••]. Seventy-eight patients received consolidation ipilimumab plus nivolumab after CCRT, and 75 patients received CCRT alone. The administration of combination ICI following CCRT resulted in a higher incidence of serious (grade 3/4) adverse events when compared to CCRT alone (62% vs. 25%, respectively) [31••]. Also, 55% of the patients (*n*=43/78) treated with consolidation ICI had their treatment discontinued due to adverse events, with a median time to ICI discontinuation of 1.7 months [31••]. Most common serious adverse events reported included pneumonitis (9%), fatigue (9%), and diarrhea (7%) [31••]. There were four deaths on study, likely attributed to the investigational drugs, specifically ileus (*n*=1) and pulmonary toxicities (*n*=3) [31••]. Potentially, one hypothesis to explain an increased incidence of adverse events and treatment discontinuation seen in the STIMULI study could be related to a supratherapeutic dose schedule of the ICI combination (ipilimumab 3 mg/kg plus nivolumab 1 mg/kg every 3 weeks), wherein pharmacologically distinct from the approved regimens in NSCLC (ipilimumab 1 mg/kg every 6 weeks and nivolumab 3 mg/kg every 2 weeks) [31••, 32, 33]. Indirect comparative analysis to the PACIFIC trial (NCT02125461) that investigated the role of durvalumab consolidation after CCRT in NSCLC, shows similar rates of pneumonitis, cough, and dyspnea when compared to the STIMULI study; however, the rate of serious pneumonitis was higher in the STIMULI study when compared to the PACIFIC trial (9% vs. 3.4%, respectively) [31••, 34]. The safety and efficacy of CCRT followed by ICI (monotherapy or combination) in patients with LS-SCLC remains under investigation in several ongoing trials (NCT03540420, NCT03703297, NCT04308785) [35–37].

Limited reported clinical data is available on the concurrent use of CCRT and ICI in LS-SCLC. In a phase I/II clinical trial (NCT02402920), 40 patients with LS-SCLC received CCRT plus pembrolizumab [38••]. The majority of patients experienced mild adverse events and the most common ones were fatigue (60%), dysphagia (58%), dyspnea (50%), esophagitis (43%), anemia (43%), cough (35%), and nausea (35%) [38••]. Grade 3/4 adverse events included neutropenia (13%), febrile neutropenia (8%), pneumonitis (8%, all grade 3), dyspnea (5%), respiratory failure (3%), and lung infection (8%) [38••]. No grade 5 toxicities were reported. Overall, this regimen was well tolerated with median PFS of 19.7 months (95% CI: 8.8–30.5) and median OS of 39.5 months (95% CI: 8.0–71.0) [38••]. Further characterization of safety signals with the use of concurrent trimodality therapy with chemotherapy, radiation, and ICI remains to be elucidated. Ongoing clinical trials are seeking to directly address these inquiries (e.g., NCT03811002, NCT04624204, NCT04602533, NCT04691063, NCT03585998) [40–44].

## Immunotherapy and chemoradiation in LS-SCLC

To date, the investigational use of concurrent immunotherapy with CCRT or consolidation ICI remains limited, with only two clinical trials reporting outcomes for LS-SCLC. [31••, 38••] Initial data on the use of concurrent pembrolizumab with CCRT followed by consolidation pembrolizumab is promising, while consolidation ipilimumab plus nivolumab after CCRT did not confer a significant benefit versus SoC observation [31••, 38••]. However, these trials have set the stage to establish clinical precedence for ICI plus CCRT, and aid in our understanding of LS-SCLC biology for ongoing clinical trials in this field.

In 2015, a phase I/II trial (NCT02402920) of concurrent pembrolizumab with CCRT followed by consolidation pembrolizumab started enrolling patients with LS-SCLC. To our knowledge, this is the only published study to date demonstrating the safety and efficacy of concurrent ICI with CCRT in patients with LS-SCLC [38••]. The phase I portion of this trial used a 3+3 design to establish the maximum tolerated dose (MTD) of pembrolizumab concurrent with CCRT for enrollment in the phase II portion. The MTD of pembrolizumab could not be determined, so the dose was set at 200 mg every 3 weeks, in accordance with studies in NSCLC [39]. As previously discussed, no concerning safety signals arose in this study [38••]. A total of 40 patients were enrolled, and at a median follow-up of 23.1 months, PFS was 19.7 months (95% CI: 8.8–30.5), and the median OS was 39.5 months (95% CI: 8.0–71.0) [38••]. Although the authors note this compares favorably to the median OS of 30 months seen in the CONVERT study [8], a phase III trial (NCT00433563) that evaluated once daily versus twice daily radiation with concurrent platinum/etoposide for LS-SCLC, cross-trial comparisons should be considered with reservation. Also, in this phase I/II trial of CCRT with concurrent and consolidative pembrolizumab, 67.5% patients (*n*=27) received PCI [38••]. Notably, their median OS was not reached, versus 39.5 months for those who did not receive PCI (HR 3.9, 95% CI: 1.1–13.6; *p*<0.05) [38••]. Whether this reflects a synergistic benefit of prevention of intracranial metastasis between immunotherapy and cranial irradiation via the abscopal effect versus selection and immortal biases is difficult to elucidate.

The STIMULI trial, a phase II study (NCT02046733), examined the use of consolidation nivolumab (1 mg/kg) plus ipilimumab (3 mg/kg) every 3 weeks after CCRT [31••]. Due to slow accrual, the trial ended recruitment prematurely. A total of 153 patients were randomized following CCRT completion to either consolidation ipilimumab plus nivolumab, versus observation [31••]. With a median follow-up of 22.4 months, the median PFS was similar between the two groups (10.7 months vs. 14.5 months, HR=1.02; 95% CI: 0.6–1.58; *p*=0.93, in the experimental vs. observation arms, respectively) [31••]. Similarly, there were no statistically significant differences in OS between the two groups (median OS not reached vs. 32.1 months in the experimental and observation arm, respectively; HR 0.95; 95% CI: 0.59–1.52; *p*=0.82) [31••]. As previously discussed, the median treatment duration in the experimental arm was 1.7 months, likely reflective of the high incidence of serious adverse events with combination ICI versus observation (G3/G4 AEs: 62% vs 25%, respectively) [31••]. Of note, a similar trial design, CheckMate 451 (NCT02538666), in ES-SCLC utilizing the same dose schedule with consolidative dual ICI with ipilimumab and nivolumab following chemotherapy did not reach its primary endpoint of OS when compared to placebo [45]. As observed in STIMULI, a large proportion of patients in the combination ICI arm in CheckMate 451 experienced treatment limiting or serious adverse events (G3/G4 AEs: 59.3%) including colitis (6.8%) and pneumonitis (5.4%) [45]. However, in CheckMate227 (NCT02477826), a Phase III trial in NSCLC utilized ipilimumab plus nivolumab combination in arm B that was safely tolerated at a dose schedule of ipilimumab 1 mg/kg every 6 weeks and nivolumab 3 mg/kg every 2 weeks [32]. The limited duration of time on investigational treatment (1.7 months) and increased toxicities with the dual ICI regimen likely obscured potential efficacy signals for activity in SCLC. Trials in LS-SCLC to evaluate different ICI combinations in this setting are ongoing and may provide answers to these questions.

## Ongoing clinical trials

As of February of 2022, ten clinical trials are actively investigating the role of ICIs in LS-SCLC. These trials vary in ICI class, mechanism of action, timing, dosing schedule, and ICI combinations. A summary of ongoing clinical trials and trials to be activated this year for LS-SCLC is listed in Table 1.

**Table 1:** Ongoing clinical trials using immunotherapy for patients with LS-SCLC

Trial (NCT #)	Estimated Enrollment	Treatment Regimen*	Phase	Outcomes	Recruitment Status	ICI Administration
NCT03585998	51	**Arm I:** Durvalumab (IV) with CCRT, followed by durvalumab (IV) for up to 2 years	II	**Primary Outcome:** PFS **Selected** **Secondary Outcomes:** OS, AEs	Unknown	Concurrent and Consolidation
ACHILES (NCT03540420)	212	**Arm I:** Atezolizumab 1200 mg (IV) Q3W after CCRT for up to 1 year **Arm II:** Observation after CCRT	II	**Primary Outcome:** 2-year OS **Selected Secondary Outcomes:** PFS, Best RR, TRAEs	Recruiting	Consolidation
ADRIATIC (NCT03703297)	728	**Arm I:** Durvalumab 1500 mg (IV) Q4W + placebo (IV) Q4W for up to 4 cycles, followed by durvalumab 1500 mg (IV) Q4W for up to 2 years **Arm II:** Durvalumab 1500 mg (IV) Q4W + tremelimumab 75 mg (IV) Q4W for up to 4 cycles, followed by durvalumab 1500 mg (IV) Q4W for up to 2 years **Arm III:** Placebo (IV) Q4W + placebo (IV) Q4W , followed by placebo (IV) Q4W for up to 2 years	III	**Primary Outcomes:** PFS and OS (Arm I) **Selected Secondary Outcomes**: ORR, PFS and OS (Arm II), OS/PFS in relation to tumor PD-L1 expression	Active, Not Recruiting	Consolidation
LU005 (NCT03811002)	506	**Arm I:** CCRT followed by observation **Arm II:** Atezolizumab (IV) Q3W with CCRT, followed by atezolizumab (IV) Q3W for up to 1 year	II/III	**Primary Outcome:** OS **Selected Secondary Outcomes:** PFS, AEs, ORR, bTMB, and tTMB	Recruiting	Concurrent and Consolidation
KEYLYNK-013 (NCT04624204)	672	**Arm A:** Pembrolizumab 200 mg (IV) Q3W with CCRT, followed by 9 cycles of pembrolizumab 400 mg (IV) Q6W + olaparib matching placebo (PO) BID for up to 1 year **Arm B:** Pembrolizumab 200 mg (IV) Q3W with CCRT, followed by 9 cycles of pembrolizumab 400 mg (IV) Q6W + olaparib (PO) 300 mg BID for up to 1 year **Arm C:** Pembrolizumab matching placebo (IV) Q3W with CCRT, followed by pembrolizumab matching placebo (IV) Q6W + olaparib matching placebo (PO) BID for up to 1 year	III	**Primary Outcomes:** PFS and OS **Selected Secondary Outcomes:** Aes, ORR, DOR In relation to PD-L1 status, ORR, DOR, PFS, and OS	Recruiting	Concurrent and Consolidation
DOLPHIN (NCT04602533)	105	**Arm I:** Durvalumab 1500 mg (IV) Q3W for 4 to 6 cycles with CCRT, followed bydurvalumab 1500 mg (IV) Q4W until disease progression or unacceptable AEs **Arm II:** CCRT for 4 cycles, followed by observation	II	**Primary Outcome:** PFS at 18 months **Selected Secondary Outcomes:** PFS, OS, ORR, DCR, AEs	Recruiting	Concurrent and Maintenance
AdvanTIG-204 (NCT04952597)	120	**Arm I:** Ociperlimab (IV) + tislelizumab (IV) with CCRT for 4 cycles, followed by ociperlimab (IV) + tislelizumab (IV) **Arm II:** Tislelizumab (IV) with CCRT for 4 cycles, followed by tislelizumab (IV) **Arm III:** CCRT for 4 cycles, followed by observation	II	**Primary Outcome:** PFS **Selected** **Secondary Outcomes:** CR, DOR, ORR, OS, Safety, Tolerability	Recruiting	Concurrent and Consolidation
NCT04691063	486	**Arm A:** SHR-1316 (IV) with CCRT **Arm B:** Placebo with CCRT	III	**Primary Outcome:** OS **Selected** **Secondary Outcomes:** None listed	Enrolling by Invitation	Concurrent
NCT05034133	20	**Arm I:** Durvalumab 1000 mg (IV) with cisplatin and etoposide for 6 cycles, followed by thoracic radiation	II	**Primary Outcome:** PFS **Selected** **Secondary Outcomes:** OS, AEs	Recruiting	Induction
ML41257 (NCT04308785)	150	**Arm A:** Atezolizumab 1200 mg (IV) Q3W + tiragolumab 600 mg (IV) Q3W for up to 17 cycles **Arm B:** Atezolizumab 1200 mg (IV) Q3W + placebo (IV) q3w for up to 17 cycles	II	**Primary Outcome:** PFS **Selected** **Secondary Outcomes:** OS, ORR, DOR, AEs	Recruiting	Consolidation
NCT04647357	60	**Arm I:** SHR-1316 (IV) Q3W	II	**Primary Outcome:** PFS **Selected** **Secondary Outcomes:** None listed	Not yet recruiting	Maintenance
NCT04189094	140	**Arm I:** Sintilimab (IV) with cisplatin or carboplatin (IV) plus etoposide (IV) Q3W for 2 cycles, followed by CCRT for 2 cycles After PCI (25 Gy in 10 fractions), sintilimab (IV) Q3W for up to 13 cycles **Arm II:** Cisplatin or carboplatin (IV) plus etoposide (IV) Q3W for 2 cycles, followed by CCRT for 2 cycles After PCI (25 Gy in 10 fractions), observation	II	**Primary Outcome:** PFS **Selected** **Secondary Outcomes:** OS, ORR	Not yet recruiting	Induction and Consolidation
NCT04418648	170	**Arm I:** Toripalimab 240 mg (IV) Q3W for up to 6 months **Arm II:** Observation	II	**Primary Outcome:** PFS **Selected** **Secondary Outcomes:** OS, ORR, DOR, AEs	Not yet recruiting	Consolidation

*Dose and frequency details are as specified on ClinicalTrials.gov. Information accessed on February 28, 2022

**Abbreviations:** *AEs* adverse events, *BID* twice a day, *bTMB* blood-based tumor mutational burden, *CR* complete response, *CCRT* concurrent chemoradiation with platinum plus etoposide, *DCR* disease control rate, *DOR* duration of response, *OS* overall survival, *ORR* Overall response rate, *PD* progressive disease, *PFS* progression-free survival, *Q2W* every 2 weeks, *Q3W* every 3 weeks, *Q4W* every 4 weeks, *Q6W* every 6 weeks, *TRAE* treatment-related adverse events, *tTMB* tissue-based tumor mutational burden; prophylactic cranial irradiation, PCI, *Gy* gray

Given the overall survival benefit with the addition of atezolizumab demonstrated with IMpower133 in ES-SCLC [11], the multi-institutional randomized phase II trial ACHILES (NCT03540420) seeks to investigate whether there is a role for integration of atezolizumab into the consolidative phase after CCRT in LS-SCLC. The study began enrolling patients in July of 2018, where patients are randomized after completion of CCRT to either receive atezolizumab 1,200 mg every 3 weeks for up to 12 months or observation [35]. Investigators expect to enroll a total of 212 patients with a primary endpoint of 2-year OS. The primary completion date is estimated for December of 2023 [35].

Soon after the activation of the ACHILES trial, the phase III, randomized ADRIATIC trial (NCT03703297) began patient enrollment in September of 2018 [36, 46]. Following CCRT completion, patients with LS-SCLC are randomized to receive either consolidation durvalumab 1500 mg plus tremelimumab 75 mg (up to four doses) every 4 weeks (Arm I), durvalumab 1500 mg plus placebo every 4 weeks (up to four doses) (Arm II), or combination placebo every 4 weeks (Arm III) followed by durvalumab single agent every 4 weeks (Arms I and II) or placebo every 4 weeks (Arm III) for up to two years. The primary endpoints of this trial are PFS and OS [36, 46]. The ADRIATIC trial has a target enrollment of 728 patients. While not actively recruiting patients, interim results are still pending with a primary outcome analysis expected by May of 2024 [36, 46].

In the context of STIMULI, in which the administration of consolidation ipilimumab plus nivolumab did not improve patients’ outcomes, the investigation of an alternative dual ICI regimen with the ADRIATIC trial remains of great interest. However, ADRIATIC does not exclusively account for parameters leading to treatment discontinuation in the STIMULI trial, such as supratherapeutic dose schedule nor does it address if ipilimumab plus nivolumab is efficacious in LS-SCLC [31••]. On the other hand, patients in the ADRIATIC trial may not experience high rates of adverse events and treatment discontinuation as the dosages of durvalumab and tremelimumab being investigated are in line with what has been used in NSCLC studies with limited dual ICI drug exposure with 4 cycles of tremelimumab [36, 46, 47]. A follow-up study to the STIMULI trial is not currently planned; however, the ADRIATIC study may elucidate a role for dual ICI blockade in patients with LS-SCLC.

Another treatment paradigm under investigation is the concurrent use of ICI during chemoradiotherapy [40, 42, 44, 48, 49]. The first study to open investigating this treatment modality was a single-arm, phase II study (NCT03585998) in Korea, which started enrolling patients in June of 2018 [44]. The target enrollment for this trial was 51 patients, where all patients receive durvalumab concurrent with chemoradiotherapy, followed by durvalumab consolidation [44]. The anticipated primary completion was set for June of 2021 with a primary endpoint of PFS; however, no results have been reported to date [44].

Next, in May of 2019, the NRG-LU005 trial began enrolling patients with LS-SCLC (NCT03811002) [40, 49]. This study is a National Cancer Institute (NCI) sponsored phase II/III randomized trial in which patients are assigned to receive CCRT followed by observation versus atezolizumab with CCRT followed by consolidation atezolizumab every 3 weeks for up to 1 year. The trial is expected to enroll 506 patients with a primary endpoint of OS. The estimated primary completion date is December of 2026 [40, 49].

Shortly after the initiation of the NRG-LU005 study, a German investigator-initiated trial (DOLPHIN, NCT04602533) began patient enrollment in October of 2020 [42, 48]. This phase II, randomized, open-label trial is assessing the efficacy and safety of concurrent durvalumab with CCRT, followed by maintenance durvalumab versus CCRT followed by observation [42, 48]. Unlike, the NRG-LU005 [40, 49], in which patients will receive consolidation ICI for up to 1 year, in the DOLPHIN study [42, 48] patients in the experimental arm will receive durvalumab 1,500 mg every 4 weeks until disease progression or unacceptable toxicities. The primary endpoint of this study is PFS at 18 months [42, 48]. The target enrollment is 105 patients with estimated primary completion date of March of 2022 [42, 48].

Of note, another phase III trial (NCT04691063) seeks to evaluate the role of the anti-PD-L1 monoclonal antibody SHR-1316 concurrent with CCRT in LS-SCLC [43]. An estimated 468 patients will be randomized to SHR-1316 with CCRT versus CCRT alone [43]. It is not stated whether consolidation SHR-1316 will be maintained after completion of CCRT. The anticipated primary completion date is May of 2025 [43].

In addition to the studies investigating ICI with CCRT or during consolidation, one study is evaluating induction ICI with chemotherapy followed by thoracic radiation (NCT05034133). This phase II trial was activated in September of 2021, and patients will be assigned to receive induction durvalumab (1000 mg IV) plus cisplatin and etoposide for six cycles followed by thoracic radiation [50]. This single arm study plans to enroll a total of 20 patients with a primary outcome of PFS [50]. The expected primary completion date is August of 2023 [50]. While sequential chemotherapy and radiotherapy is not the SoC for LS-SCLC, this approach is routinely used in frail patients that cannot tolerate concurrent treatment modalities due to toxicities or preexisting comorbidities. If efficacious, a sequential therapeutic approach of ICI with chemotherapy followed by radiotherapy would offer an alternative treatment modality for LS-SCLC.

Novel therapeutic combinations including the concomitant use of PARP inhibitors (KEYLYNK-013) or anti-TIGIT monoclonal antibodies (AdvanTIG-204, ML41257) are being actively investigated with immunotherapy in LS-SCLC [37, 41, 51]. KEYLYNK-013 (NCT04624204) [41] utilized safety data from a phase I/II trial (NCT02402920) using pembrolizumab with CCRT followed by pembrolizumab consolidation to further investigate the role of olaparib in this treatment modality [38••]. Specifically, in this phase III, randomized, placebo-controlled, double-blind trial, 672 patients will be randomized to one of three arms: pembrolizumab with CCRT followed by consolidation pembrolizumab and olaparib placebo (Arm A), pembrolizumab with CCRT followed by consolidation pembrolizumab and olaparib (Arm B), or pembrolizumab placebo with CCRT followed by consolidation pembrolizumab placebo and olaparib placebo (Arm C) [41]. The primary endpoints of this study are PFS and OS, and the estimated primary completion is expected by October of 2027 [41].

The rationale behind the use of PARP inhibitors for SCLC derives from the high expression of PARP1 in these cancer cells [52]. Preclinical evidence supports a synergistic effect between PARP inhibitors and ICI in SCLC [53]; however, currently available data is mixed [54, 55]. One study examined the combination of durvalumab and olaparib for relapsed SCLC [54]. Of the 19 evaluable patients, there were two patients with partial or complete response, and four patients with stable disease with confirmed response or stable disease for >8 months [54]. Most patients experienced at least one adverse event, including anemia (80%), lymphopenia (60%), and leukopenia (50%); 45% of patients (*n*=9) experienced a serious adverse event [54]. A second study, MEDIOLA (NCT02734004) [55], evaluated the combination of olaparib and durvalumab in relapsed SCLC. Among the 38 evaluable patients, 29% had disease control at 12 weeks with two confirmed responses (one complete and one partial response) [55]. The combination was relatively well tolerated, and the most common serious adverse events were anemia (34.2%), hyponatremia (10.5%), and lymphopenia (10.5%) [55]. KEYLYNK-013 (NCT04624204), contrary to the previously mentioned trials in patients with relapsed SCLC, seeks to investigate the role of combinatory PARP inhibition (PARPi) with ICIs in the consolidative setting for LS-SCLC [41]. Whether PARPi can deepen clinical response to ICIs in LS-SCLC is anxiously awaited.

A new therapeutic approach for LS-SCLC includes the combination of an anti-TGIT monoclonal antibody with immune checkpoint inhibitors, such as anti-PD-L1, anti-PD1, and anti-CTLA4 antibodies. The phase II, multicenter, open-label Chinese study, AdvanTIG-204 (NCT04952597) started enrolling patients in July of 2021 [51]. This trial seeks to investigate the novel combination of the PD-1 inhibitor tislelizumab and the anti-TIGIT monoclonal antibody ociperlimab [51]. An estimated 120 patients will be enrolled and randomized to one of three arms: tislelizumab plus ociperlimab with CCRT followed by tislelizumab plus ociperlimab consolidation, tislelizumab with CCRT followed by tislelizumab consolidation, or CCRT alone followed by observation [51]. The primary endpoint of this study is PFS and the estimated primary completion date is March of 2024. Similarly, the phase II trial ML41257 (NCT04308785) is evaluating consolidation atezolizumab and the anti-TIGIT antibody tiragolumab vs. atezolizumab consolidation alone [37]. An estimated 150 patients will be enrolled, beginning in December of 2021 [37]. The primary endpoint of this study is PFS, and the estimated primary completion date is June of 2024 [37].

TIGIT, a co-inhibitory signaling molecule on the surface of a variety of T cells including CD8+ tumor infiltrating lymphocytes and regulatory T cells (Tregs), may dampen the anti-tumor immune response [56]. CITYSCAPE (NCT03563716) evaluated atezolizumab with or without tiragolumab in chemotherapy-naïve patients with stage IV NSCLC [57]. The combination of atezolizumab plus tiragolumab improved PFS compared to atezolizumab plus placebo (median PFS 5.4 vs 3.6 months, respectively, HR 0.57; 95% CI: 0.37–0.90). However, in a subgroup analysis, significant benefit in mPFS is enriched in patients whose tumor had a high PD-L1 expression (TPS ≥50%). Serious adverse events rates were similar between both treatment groups occuring in 14.9% with atezolizumab plus tiragolumab versus 19.1% with atezolizumab plus placebo [57]. It will be important to assess whether tumoral PD-L1 expression will impact outcomes in patients with LS-SCLC treated with anti-CTLA4 and anti-TIGIT monoclonal antibodies.

Upcoming trials including the assessment of maintenance SHR-1316, induction and consolidation sintilimab, and toripalimab consolidation (NCT04647357, NCT04189094, NCT04418648) are underway, beginning in 2022 [58–60].

## Conclusions

An abundance of preclinical data suggests a synergistic benefit between ICI and radiation therapy [16, 18–20]. These findings led to early phase studies in LS-SCLC and other malignancies, with a generally tolerable safety profile. CCRT with concurrent and consolidative pembrolizumab had favorable tolerability and activity in LS-SCLC [38••]. However, the phase II STIMULI trial did not meet its primary endpoint of PFS, and consolidative ipilimumab with nivolumab was not well tolerated [31••]. Given the mixed data, the ten ongoing trials evaluating the safety and efficacy of immunotherapy, CCRT, and novel agents such as PARP inhibitors or anti-TIGIT antibodies for LS-SCLC will serve as a catalyst to defining the role of ICIs within the field of thoracic oncology. Critical questions regarding the impact of tumoral PD-L1 expression and the timing of ICI administration with CCRT on patient outcomes remains to be answered. Together, these trials continue to move the benchmark forward and offer hope for improvement in curative approaches for patients with limited stage SCLC.
